# 5,7-Bis(benz­yloxy)-2-[4-(benz­yloxy)phen­yl]-4*H*-chromen-4-one

**DOI:** 10.1107/S1600536809051678

**Published:** 2009-12-04

**Authors:** Xu-Hong Ren, Wei-Jia Xie, Chao Ma, Jing-Jing Wang, Mao-Sheng Cheng

**Affiliations:** a School of Pharmaceutical Engineering, Shenyang Pharmaceutical University, Mail Box 40, 103 Wenhua Road, Shenhe District, Shenyang 110016, People’s Republic of China; b School of Pharmaceutical Sciences, Kinki University, 3-4-1 Kowakae, Higashi-osaka, Osaka 577-8502, Japan

## Abstract

In the title compound, C_36_H_28_O_5_, the terminal benzene rings are twisted at dihedral angles of 6.75 (12), 70.86 (14) and 82.02 (12)° with the respect to the central plana r[maximum deviation = 0.070 (3) Å] chromen-4-one ring system. In the crystal structure, π–π stacking is observed between parallel benzene rings of adjacent mol­ecules [centroid–centroid distance = 3.7459 (16) Å].

## Related literature

For general background to the biological effects of flavones, see: Formica & Regelson (1995 ▶); Medina *et al.* (1998 ▶); Cotelle *et al.* (1992 ▶). For a related structure, see: Waller *et al.* (2003 ▶).
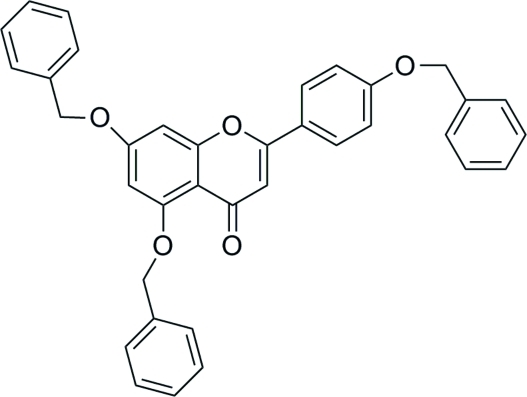


## Experimental

### 

#### Crystal data

C_36_H_28_O_5_
*M* *_r_* = 540.58
Triclinic, 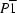

*a* = 7.3176 (12) Å
*b* = 12.818 (2) Å
*c* = 14.933 (2) Å
α = 82.542 (3)°
β = 83.861 (3)°
γ = 85.600 (3)°
*V* = 1378.0 (4) Å^3^
*Z* = 2
Mo *K*α radiation
μ = 0.09 mm^−1^
*T* = 293 K
0.35 × 0.22 × 0.08 mm

#### Data collection

Bruker SMART APEX CCD area-detector diffractometer
7198 measured reflections
4786 independent reflections
2505 reflections with *I* > 2σ(*I*)
*R*_int_ = 0.021

#### Refinement

*R*[*F* ^2^ > 2σ(*F* ^2^)] = 0.054
*wR*(*F* ^2^) = 0.140
*S* = 0.99
4786 reflections
370 parameters
H-atom parameters constrained
Δρ_max_ = 0.13 e Å^−3^
Δρ_min_ = −0.14 e Å^−3^

### 

Data collection: *SMART* (Bruker, 1997 ▶); cell refinement: *SAINT* (Bruker, 1999 ▶); data reduction: *SAINT*; program(s) used to solve structure: *SHELXS97* (Sheldrick, 2008 ▶); program(s) used to refine structure: *SHELXL97* (Sheldrick, 2008 ▶); molecular graphics: *ORTEP-3 for Windows* (Farrugia, 1997 ▶); software used to prepare material for publication: *SHELXL97*.

## Supplementary Material

Crystal structure: contains datablocks I, global. DOI: 10.1107/S1600536809051678/xu2692sup1.cif

Structure factors: contains datablocks I. DOI: 10.1107/S1600536809051678/xu2692Isup2.hkl

Additional supplementary materials:  crystallographic information; 3D view; checkCIF report

## Figures and Tables

**Fig. 1.**
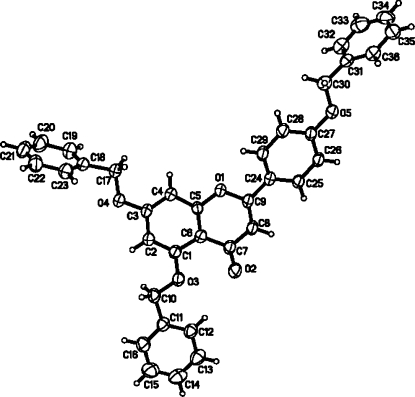
The structure of the title compound, showing 30% probability displacement ellipsoids and the atom-numbering scheme.

## supplementary crystallographic information

### Comment

Flavones are among the most ubiquitous groups of polyphenolic compounds in foods of plant origin. As integral constituents of the diet, they may exert a wide range of beneficial effects on human health, including protection against cardiovascular disease, certain forms of cancer (Formica & Regelson 1995) and modulatory activities at GABA-A receptors (Medina *et al.*, 1998). Flavones likely produce such biological effects through their free radical-scavenging antioxidative activities and metal ion-chelating a bilities (Cotelle *et al.*1992). Some flavones are more potent than ascorbic acid and tocopherols in scavenging reactive oxygen species. The title compound was crystallized as part of an ongoing structure-activity study to determine the properties of those compounds that confer this activity in order to aid the design of more active compounds.

The molecular is shown in shown in Fig. 1, the bond lengths and angles are within normal ranges. The bond length of the carbonyl group C7=O2 of 1.228 Å is somewhat longer than typical carbonyl bond. This may be due to the fact that atom O2 participate in intermolecular Van der Waals forces. And the bond lengths of C1—O3, C3—O4 and C27—O5 are 1.353 Å, 1.369 Å and 1.375 Å, respectively. In the crystal structure π-π stacking is observed between parallel C24-benzene and C24^i^-benzene rings of adjacent molecules [centroids distance 3.7459 (16) Å; symmetry code: (i) 2-x, 1-y, 2-z].

### Experimental

*E*-3-(4-Phenoxyphenyl)-1-(2,4-bisphenoxy-6-hydroxyphenyl) propenone (2 g, 3.40 mmol) was dissolved in DMSO (40 ml). I_2_ (96 mg, 0.377 mmol) was added to the solution. The reaction mixture was heated at 400 K for 2 h under N_2_, then coolded to room temperature. The mixture was pured to 200 ml 1M HCl solution. The aqueous layer was extracted with ethyl acetate. The organic layer was washed with NaHCO_3_ solution, watre and brine, and dried by MgSO_4_ and concentrated under diminished pressure. The residue was purified by flash chromatography on a silica gel column (elutant: hexanesethyl /acetate, 4:1). Single crystals suitable for X-ray diffraction were obtained by solw evaporation of a dilute solution of the title compound in methanol/dichloromethane (1:5).

### Refinement

The H atoms were placed in calculated positions and refined in riding mode with U_iso_(H) = 1.2U_eq_(C).

### Crystal data

**Table:**

C_36_H_28_O_5_	*Z* = 2
*M**_r_* = 540.58	*F*(000) = 568
Triclinic, *P*1	*D*_x_ = 1.303 Mg m^−^^3^
Hall symbol: -P 1	Mo *K*α radiation, λ = 0.71073 Å
*a* = 7.3176 (12) Å	Cell parameters from 1081 reflections
*b* = 12.818 (2) Å	θ = 2.2–21.3°
*c* = 14.933 (2) Å	µ = 0.09 mm^−^^1^
α = 82.542 (3)°	*T* = 293 K
β = 83.861 (3)°	Platelet, colorless
γ = 85.600 (3)°	0.35 × 0.22 × 0.08 mm
*V* = 1378.0 (4) Å^3^	

### Data collection

**Table:**

Bruker SMART APEX CCD area-detector diffractometer	2505 reflections with *I* > 2σ(*I*)
Radiation source: fine-focus sealed tube	*R*_int_ = 0.021
graphite	θ_max_ = 25.0°, θ_min_ = 1.4°
φ and ω scans	*h* = −8→7
7198 measured reflections	*k* = −15→14
4786 independent reflections	*l* = −17→15

### Refinement

**Table:**

Refinement on *F*^2^	Primary atom site location: structure-invariant direct methods
Least-squares matrix: full	Secondary atom site location: difference Fourier map
*R*[*F*^2^ > 2σ(*F*^2^)] = 0.054	Hydrogen site location: inferred from neighbouring sites
*wR*(*F*^2^) = 0.140	H-atom parameters constrained
*S* = 0.99	*w* = 1/[σ^2^(*F*_o_^2^) + (0.0563*P*)^2^ + 0.0156*P*] where *P* = (*F*_o_^2^ + 2*F*_c_^2^)/3
4786 reflections	(Δ/σ)_max_ < 0.001
370 parameters	Δρ_max_ = 0.13 e Å^−^^3^
0 restraints	Δρ_min_ = −0.14 e Å^−^^3^

### Special details

**Table:**

Geometry. All e.s.d.'s (except the e.s.d. in the dihedral angle between two l.s. planes) are estimated using the full covariance matrix. The cell e.s.d.'s are taken into account individually in the estimation of e.s.d.'s in distances, angles and torsion angles; correlations between e.s.d.'s in cell parameters are only used when they are defined by crystal symmetry. An approximate (isotropic) treatment of cell e.s.d.'s is used for estimating e.s.d.'s involving l.s. planes.
Refinement. Refinement of *F*^2^ against ALL reflections. The weighted *R*-factor *wR* and goodness of fit *S* are based on *F*^2^, conventional *R*-factors *R* are based on *F*, with *F* set to zero for negative *F*^2^. The threshold expression of *F*^2^ > σ(*F*^2^) is used only for calculating *R*-factors(gt) *etc*. and is not relevant to the choice of reflections for refinement. *R*-factors based on *F*^2^ are statistically about twice as large as those based on *F*, and *R*- factors based on ALL data will be even larger.

### Fractional atomic coordinates and isotropic or equivalent isotropic displacement parameters (Å^2^)

**Table:**

	*x*	*y*	*z*	*U*_iso_*/*U*_eq_	
C1	0.4399 (4)	0.5313 (2)	0.65974 (17)	0.0615 (7)	
C2	0.3089 (3)	0.6140 (2)	0.65568 (17)	0.0637 (7)	
H2	0.2078	0.6118	0.6233	0.076*	
C3	0.3274 (4)	0.7009 (2)	0.69977 (19)	0.0650 (8)	
C4	0.4748 (3)	0.7066 (2)	0.74818 (18)	0.0666 (8)	
H4	0.4882	0.7653	0.7770	0.080*	
C5	0.6029 (3)	0.6210 (2)	0.75215 (17)	0.0569 (7)	
C6	0.5971 (3)	0.5329 (2)	0.70836 (17)	0.0578 (7)	
C7	0.7481 (4)	0.4513 (2)	0.71387 (19)	0.0646 (7)	
C8	0.8810 (4)	0.4680 (2)	0.77456 (18)	0.0649 (7)	
H8	0.9751	0.4161	0.7845	0.078*	
C9	0.8767 (3)	0.5535 (2)	0.81716 (17)	0.0567 (7)	
C10	0.2776 (4)	0.4369 (2)	0.56895 (18)	0.0717 (8)	
H10A	0.2759	0.4939	0.5195	0.086*	
H10B	0.1631	0.4428	0.6078	0.086*	
C11	0.2968 (4)	0.3326 (2)	0.53217 (17)	0.0658 (7)	
C12	0.4499 (4)	0.2647 (2)	0.53946 (18)	0.0764 (8)	
H12	0.5462	0.2817	0.5697	0.092*	
C13	0.4618 (5)	0.1707 (3)	0.5019 (2)	0.0919 (10)	
H13	0.5659	0.1248	0.5073	0.110*	
C14	0.3212 (6)	0.1451 (3)	0.4571 (2)	0.1017 (11)	
H14	0.3297	0.0821	0.4316	0.122*	
C15	0.1680 (6)	0.2126 (3)	0.4499 (2)	0.1067 (12)	
H15	0.0720	0.1956	0.4194	0.128*	
C16	0.1558 (4)	0.3050 (3)	0.4874 (2)	0.0909 (10)	
H16	0.0505	0.3500	0.4826	0.109*	
C17	0.1970 (4)	0.8678 (2)	0.7378 (2)	0.0819 (9)	
H17A	0.3049	0.9057	0.7139	0.098*	
H17B	0.2055	0.8452	0.8018	0.098*	
C18	0.0252 (4)	0.9384 (2)	0.7251 (2)	0.0664 (8)	
C19	0.0278 (4)	1.0262 (2)	0.6621 (2)	0.0863 (10)	
H19	0.1349	1.0402	0.6244	0.104*	
C20	−0.1269 (6)	1.0941 (3)	0.6540 (2)	0.1007 (11)	
H20	−0.1230	1.1539	0.6115	0.121*	
C21	−0.2839 (5)	1.0739 (3)	0.7077 (2)	0.0919 (11)	
H21	−0.3878	1.1199	0.7021	0.110*	
C22	−0.2905 (4)	0.9869 (3)	0.7696 (2)	0.0881 (10)	
H22	−0.3990	0.9730	0.8063	0.106*	
C23	−0.1360 (4)	0.9187 (2)	0.7783 (2)	0.0807 (9)	
H23	−0.1414	0.8588	0.8207	0.097*	
C24	1.0066 (3)	0.5790 (2)	0.87816 (17)	0.0545 (7)	
C25	1.1804 (3)	0.5272 (2)	0.88121 (17)	0.0605 (7)	
H25	1.2117	0.4714	0.8473	0.073*	
C26	1.3063 (4)	0.5567 (2)	0.93301 (17)	0.0638 (7)	
H26	1.4220	0.5213	0.9335	0.077*	
C27	1.2622 (4)	0.6387 (2)	0.98449 (17)	0.0610 (7)	
C28	1.0861 (4)	0.6875 (2)	0.98691 (18)	0.0695 (8)	
H28	1.0524	0.7401	1.0240	0.083*	
C29	0.9617 (4)	0.6573 (2)	0.93393 (18)	0.0656 (7)	
H29	0.8440	0.6905	0.9356	0.079*	
C30	1.3635 (4)	0.7560 (2)	1.0795 (2)	0.0817 (9)	
H30A	1.2865	0.7359	1.1351	0.098*	
H30B	1.2981	0.8127	1.0439	0.098*	
C31	1.5407 (4)	0.7924 (2)	1.1012 (2)	0.0676 (8)	
C32	1.6274 (5)	0.8700 (3)	1.0444 (2)	0.0886 (10)	
H32	1.5782	0.8973	0.9907	0.106*	
C33	1.7863 (6)	0.9085 (3)	1.0654 (3)	0.1061 (12)	
H33	1.8439	0.9613	1.0263	0.127*	
C34	1.8587 (5)	0.8682 (4)	1.1447 (4)	0.1072 (13)	
H34	1.9651	0.8941	1.1598	0.129*	
C35	1.7749 (5)	0.7903 (3)	1.2013 (3)	0.0928 (10)	
H35	1.8257	0.7624	1.2544	0.111*	
C36	1.6151 (4)	0.7525 (2)	1.1804 (2)	0.0796 (9)	
H36	1.5576	0.7000	1.2198	0.096*	
O1	0.7420 (2)	0.63213 (13)	0.80484 (12)	0.0647 (5)	
O2	0.7642 (3)	0.37370 (17)	0.67264 (15)	0.0968 (7)	
O3	0.4297 (2)	0.44352 (14)	0.61951 (12)	0.0745 (6)	
O4	0.1888 (2)	0.77832 (14)	0.69079 (13)	0.0772 (6)	
O5	1.4013 (2)	0.66769 (14)	1.02937 (13)	0.0767 (6)	

### Atomic displacement parameters (Å^2^)

**Table:**

	*U*^11^	*U*^22^	*U*^33^	*U*^12^	*U*^13^	*U*^23^
C1	0.0644 (18)	0.0570 (18)	0.0630 (18)	0.0071 (15)	−0.0114 (15)	−0.0095 (14)
C2	0.0628 (18)	0.0577 (18)	0.0724 (19)	0.0051 (15)	−0.0217 (14)	−0.0080 (14)
C3	0.0576 (17)	0.0521 (17)	0.084 (2)	0.0097 (15)	−0.0182 (15)	−0.0008 (15)
C4	0.0617 (18)	0.0525 (17)	0.087 (2)	0.0054 (15)	−0.0198 (16)	−0.0087 (14)
C5	0.0465 (16)	0.0557 (17)	0.0681 (18)	−0.0016 (14)	−0.0129 (14)	−0.0010 (14)
C6	0.0569 (17)	0.0540 (17)	0.0605 (17)	0.0012 (14)	−0.0054 (14)	−0.0028 (13)
C7	0.0580 (18)	0.0626 (19)	0.072 (2)	0.0110 (15)	−0.0102 (15)	−0.0101 (15)
C8	0.0578 (17)	0.0625 (19)	0.0726 (19)	0.0160 (14)	−0.0137 (15)	−0.0076 (15)
C9	0.0478 (16)	0.0530 (17)	0.0656 (18)	0.0075 (14)	−0.0063 (14)	0.0016 (14)
C10	0.0716 (19)	0.076 (2)	0.0697 (19)	0.0089 (16)	−0.0236 (16)	−0.0123 (15)
C11	0.078 (2)	0.0660 (19)	0.0535 (17)	0.0057 (17)	−0.0148 (15)	−0.0061 (14)
C12	0.091 (2)	0.071 (2)	0.0679 (19)	0.0125 (18)	−0.0205 (16)	−0.0140 (16)
C13	0.120 (3)	0.081 (2)	0.075 (2)	0.018 (2)	−0.014 (2)	−0.0175 (18)
C14	0.160 (4)	0.080 (2)	0.069 (2)	−0.002 (3)	−0.021 (2)	−0.0232 (18)
C15	0.140 (3)	0.099 (3)	0.092 (3)	−0.011 (3)	−0.043 (2)	−0.024 (2)
C16	0.098 (3)	0.091 (3)	0.090 (2)	0.010 (2)	−0.041 (2)	−0.020 (2)
C17	0.078 (2)	0.066 (2)	0.109 (2)	0.0175 (16)	−0.0397 (18)	−0.0230 (18)
C18	0.068 (2)	0.0559 (18)	0.079 (2)	0.0085 (16)	−0.0256 (17)	−0.0155 (15)
C19	0.083 (2)	0.083 (2)	0.089 (2)	0.0090 (19)	−0.0125 (18)	−0.0008 (19)
C20	0.116 (3)	0.077 (2)	0.105 (3)	0.024 (2)	−0.028 (2)	0.005 (2)
C21	0.099 (3)	0.085 (3)	0.093 (3)	0.038 (2)	−0.032 (2)	−0.026 (2)
C22	0.072 (2)	0.105 (3)	0.088 (2)	0.014 (2)	−0.0149 (18)	−0.023 (2)
C23	0.087 (2)	0.069 (2)	0.086 (2)	0.0047 (19)	−0.0244 (19)	−0.0023 (16)
C24	0.0492 (16)	0.0534 (16)	0.0576 (17)	0.0075 (13)	−0.0050 (13)	−0.0003 (13)
C25	0.0590 (17)	0.0578 (17)	0.0649 (18)	0.0097 (14)	−0.0118 (14)	−0.0120 (13)
C26	0.0600 (17)	0.0620 (18)	0.0685 (19)	0.0157 (15)	−0.0137 (15)	−0.0101 (15)
C27	0.0597 (18)	0.0608 (18)	0.0623 (18)	0.0112 (15)	−0.0166 (14)	−0.0071 (14)
C28	0.0677 (19)	0.0695 (19)	0.072 (2)	0.0157 (16)	−0.0138 (16)	−0.0195 (15)
C29	0.0576 (17)	0.0646 (19)	0.0727 (19)	0.0156 (14)	−0.0132 (15)	−0.0072 (15)
C30	0.079 (2)	0.073 (2)	0.097 (2)	0.0116 (17)	−0.0174 (18)	−0.0276 (18)
C31	0.0644 (19)	0.0577 (19)	0.082 (2)	0.0105 (16)	−0.0081 (17)	−0.0223 (16)
C32	0.091 (3)	0.079 (2)	0.094 (2)	0.005 (2)	−0.005 (2)	−0.011 (2)
C33	0.091 (3)	0.081 (3)	0.144 (4)	−0.015 (2)	0.018 (3)	−0.028 (3)
C34	0.076 (3)	0.107 (3)	0.153 (4)	0.000 (2)	−0.008 (3)	−0.077 (3)
C35	0.085 (3)	0.101 (3)	0.100 (3)	0.016 (2)	−0.023 (2)	−0.038 (2)
C36	0.081 (2)	0.072 (2)	0.087 (2)	0.0076 (18)	−0.0140 (18)	−0.0186 (17)
O1	0.0524 (10)	0.0543 (11)	0.0878 (13)	0.0077 (9)	−0.0182 (10)	−0.0081 (9)
O2	0.0871 (15)	0.0890 (16)	0.1244 (18)	0.0318 (12)	−0.0368 (13)	−0.0515 (14)
O3	0.0747 (13)	0.0691 (13)	0.0845 (14)	0.0159 (10)	−0.0314 (11)	−0.0211 (11)
O4	0.0724 (13)	0.0595 (12)	0.1057 (15)	0.0187 (10)	−0.0378 (11)	−0.0223 (11)
O5	0.0711 (13)	0.0715 (13)	0.0933 (14)	0.0202 (10)	−0.0288 (11)	−0.0291 (11)

### Geometric parameters (Å, °)

**Table:**

C1—O3	1.352 (3)	C18—C19	1.370 (4)
C1—C2	1.374 (3)	C18—C23	1.371 (4)
C1—C6	1.427 (3)	C19—C20	1.380 (4)
C2—C3	1.388 (3)	C19—H19	0.9300
C2—H2	0.9300	C20—C21	1.351 (4)
C3—O4	1.368 (3)	C20—H20	0.9300
C3—C4	1.373 (3)	C21—C22	1.353 (4)
C4—C5	1.387 (3)	C21—H21	0.9300
C4—H4	0.9300	C22—C23	1.382 (4)
C5—O1	1.378 (3)	C22—H22	0.9300
C5—C6	1.382 (3)	C23—H23	0.9300
C6—C7	1.463 (3)	C24—C29	1.384 (3)
C7—O2	1.228 (3)	C24—C25	1.392 (3)
C7—C8	1.443 (4)	C25—C26	1.368 (3)
C8—C9	1.334 (3)	C25—H25	0.9300
C8—H8	0.9300	C26—C27	1.380 (3)
C9—O1	1.363 (3)	C26—H26	0.9300
C9—C24	1.466 (3)	C27—O5	1.375 (3)
C10—O3	1.424 (3)	C27—C28	1.388 (3)
C10—C11	1.501 (4)	C28—C29	1.375 (3)
C10—H10A	0.9700	C28—H28	0.9300
C10—H10B	0.9700	C29—H29	0.9300
C11—C12	1.370 (3)	C30—O5	1.430 (3)
C11—C16	1.379 (4)	C30—C31	1.492 (4)
C12—C13	1.387 (4)	C30—H30A	0.9700
C12—H12	0.9300	C30—H30B	0.9700
C13—C14	1.368 (4)	C31—C32	1.370 (4)
C13—H13	0.9300	C31—C36	1.376 (4)
C14—C15	1.368 (4)	C32—C33	1.378 (4)
C14—H14	0.9300	C32—H32	0.9300
C15—C16	1.368 (4)	C33—C34	1.372 (5)
C15—H15	0.9300	C33—H33	0.9300
C16—H16	0.9300	C34—C35	1.360 (5)
C17—O4	1.428 (3)	C34—H34	0.9300
C17—C18	1.505 (3)	C35—C36	1.380 (4)
C17—H17A	0.9700	C35—H35	0.9300
C17—H17B	0.9700	C36—H36	0.9300
			
O3—C1—C2	123.3 (2)	C18—C19—H19	119.7
O3—C1—C6	115.5 (2)	C20—C19—H19	119.7
C2—C1—C6	121.2 (3)	C21—C20—C19	120.2 (3)
C1—C2—C3	120.0 (3)	C21—C20—H20	119.9
C1—C2—H2	120.0	C19—C20—H20	119.9
C3—C2—H2	120.0	C20—C21—C22	120.2 (3)
O4—C3—C4	123.8 (3)	C20—C21—H21	119.9
O4—C3—C2	114.8 (2)	C22—C21—H21	119.9
C4—C3—C2	121.5 (2)	C21—C22—C23	119.9 (3)
C3—C4—C5	117.0 (3)	C21—C22—H22	120.0
C3—C4—H4	121.5	C23—C22—H22	120.0
C5—C4—H4	121.5	C18—C23—C22	120.7 (3)
O1—C5—C6	122.0 (2)	C18—C23—H23	119.6
O1—C5—C4	112.9 (2)	C22—C23—H23	119.6
C6—C5—C4	125.1 (2)	C29—C24—C25	117.4 (2)
C5—C6—C1	115.2 (2)	C29—C24—C9	121.0 (2)
C5—C6—C7	119.2 (2)	C25—C24—C9	121.6 (2)
C1—C6—C7	125.6 (3)	C26—C25—C24	121.4 (3)
O2—C7—C8	121.1 (2)	C26—C25—H25	119.3
O2—C7—C6	124.9 (3)	C24—C25—H25	119.3
C8—C7—C6	114.0 (3)	C25—C26—C27	120.3 (2)
C9—C8—C7	123.8 (2)	C25—C26—H26	119.9
C9—C8—H8	118.1	C27—C26—H26	119.9
C7—C8—H8	118.1	O5—C27—C26	116.3 (2)
C8—C9—O1	120.5 (2)	O5—C27—C28	124.3 (3)
C8—C9—C24	128.1 (2)	C26—C27—C28	119.4 (3)
O1—C9—C24	111.4 (2)	C29—C28—C27	119.4 (3)
O3—C10—C11	108.4 (2)	C29—C28—H28	120.3
O3—C10—H10A	110.0	C27—C28—H28	120.3
C11—C10—H10A	110.0	C28—C29—C24	121.9 (2)
O3—C10—H10B	110.0	C28—C29—H29	119.0
C11—C10—H10B	110.0	C24—C29—H29	119.0
H10A—C10—H10B	108.4	O5—C30—C31	109.2 (2)
C12—C11—C16	118.6 (3)	O5—C30—H30A	109.8
C12—C11—C10	122.9 (3)	C31—C30—H30A	109.8
C16—C11—C10	118.6 (3)	O5—C30—H30B	109.8
C11—C12—C13	120.2 (3)	C31—C30—H30B	109.8
C11—C12—H12	119.9	H30A—C30—H30B	108.3
C13—C12—H12	119.9	C32—C31—C36	118.9 (3)
C14—C13—C12	120.4 (3)	C32—C31—C30	120.2 (3)
C14—C13—H13	119.8	C36—C31—C30	120.9 (3)
C12—C13—H13	119.8	C31—C32—C33	121.2 (3)
C15—C14—C13	119.5 (3)	C31—C32—H32	119.4
C15—C14—H14	120.2	C33—C32—H32	119.4
C13—C14—H14	120.2	C34—C33—C32	119.3 (4)
C14—C15—C16	120.1 (3)	C34—C33—H33	120.3
C14—C15—H15	120.0	C32—C33—H33	120.3
C16—C15—H15	120.0	C35—C34—C33	120.1 (4)
C15—C16—C11	121.2 (3)	C35—C34—H34	120.0
C15—C16—H16	119.4	C33—C34—H34	120.0
C11—C16—H16	119.4	C34—C35—C36	120.5 (4)
O4—C17—C18	108.5 (2)	C34—C35—H35	119.8
O4—C17—H17A	110.0	C36—C35—H35	119.8
C18—C17—H17A	110.0	C31—C36—C35	120.1 (3)
O4—C17—H17B	110.0	C31—C36—H36	120.0
C18—C17—H17B	110.0	C35—C36—H36	120.0
H17A—C17—H17B	108.4	C9—O1—C5	120.0 (2)
C19—C18—C23	118.2 (3)	C1—O3—C10	118.6 (2)
C19—C18—C17	120.7 (3)	C3—O4—C17	117.2 (2)
C23—C18—C17	121.0 (3)	C27—O5—C30	117.8 (2)
C18—C19—C20	120.7 (3)		
